# A critical review and meta-analysis of the unconscious thought effect in medical decision making

**DOI:** 10.3389/fpsyg.2015.00636

**Published:** 2015-05-19

**Authors:** Miguel A. Vadillo, Olga Kostopoulou, David R. Shanks

**Affiliations:** ^1^Department of Primary Care and Public Health Sciences, King’s College LondonLondon, UK; ^2^Division of Psychology and Language Sciences, University College LondonLondon, UK

**Keywords:** Bayes factors, deliberation without attention, medical decision making, meta-analysis, unconscious thought effect

## Abstract

Based on research on the increasingly popular unconscious thought effect (UTE), it has been suggested that physicians might make better diagnostic decisions after a period of distraction than after an equivalent amount of time of conscious deliberation. However, published attempts to demonstrate the UTE in medical decision making have yielded inconsistent results. In the present study, we report the results of a meta-analysis of all the available evidence on the UTE in medical decisions made by expert and novice clinicians. The meta-analysis failed to find a significant contribution of unconscious thought (UT) to the accuracy of medical decisions. This result cannot be easily attributed to any of the potential moderators of the UTE that have been discussed in the literature. Furthermore, a Bayes factor analysis shows that most experimental conditions provide positive support for the null hypothesis, suggesting that these null results do not reflect a simple lack of statistical power. We suggest ways in which new studies could usefully provide further evidence on the UTE. Unless future research shows otherwise, the recommendation of using UT to improve medical decisions lacks empirical support.

If your physician told you that instead of thinking logically and analytically about each case she prefers to rely on intuition and unconscious thought (UT) to make her diagnostic decisions, you would probably express some concern. Yet, she would be following the recommendations of an increasing number of scholars who advise practitioners and patients to rely on unconscious processes to make complex medical decisions (e.g., de Vries et al., 2010; Dhaliwal, 2010; Trowbridge et al., 2013; Manigault et al., 2015). This suggestion is also in line with the current enthusiasm in the media and in popular science books for intuition and unconscious mental processes (Gladwell, 2005; Gigerenzer, 2007; Kahneman, 2011).

Much of this interest stems from research on an intriguing phenomenon known as the UT effect (UTE, sometimes referred to as the deliberation-without-attention effect). In a typical UTE experiment, participants are exposed to a complex decision involving many variables, such as choosing between consumer products based on long lists of features of each product. After reading this information, some participants are asked to spend a few minutes thinking about it before making a decision, while other participants are instructed to spend an equal amount of time performing a distracting task, such as solving anagrams or word-search puzzles. The crucial result of these experiments is that, under some conditions, participants seem to make better decisions after the distracting task than after conscious deliberation. Most importantly, this advantage of UT over conscious thought (CT) is mainly observed for complex tasks (Dijksterhuis, 2004; Dijksterhuis et al., 2006). According to the theoretical framework proposed by these researchers, this phenomenon occurs because, unlike conscious thinking, the unconscious is not limited by working memory constraints and, consequently, is able to deal with larger amounts of information (Dijksterhuis and Nordgren, 2006). This feature of UT makes it ideal for complex decisions involving multiple cues and options.

Since the publication of the seminal UTE experiments in *Science*, researchers have shown some skepticism about the relevance of these findings for decision making in real life, particularly in the domain of health-related decisions (Bekker, 2006). However, a subsequent study by de Vries et al. (2010) suggested that these concerns might be unjustified. Clinical psychology students were presented with two complex case descriptions from the DSM-IV casebook, each comprising 2–3 paragraphs of text summarizing a patient’s case notes. After reading the descriptions, one group of participants was instructed to think about this information for 4 min, while the other group was asked to spend 4 min solving a word-finding puzzle before diagnosing. The authors found that the diagnoses of the latter group were significantly more accurate (i.e., in agreement with the diagnoses provided by the DSM-IV casebook) than the diagnoses of the former group, thereby providing the first demonstration of the UTE in medical decision making.

Subsequent attempts to find a UT advantage in medical decisions have yielded inconsistent results, though. Mamede et al. (2010) conducted a larger study, involving not only medical students but also physicians, where they manipulated the complexity of the diagnostic decision and used two different control conditions for assessing the UT advantage. Participants read summaries of real cases and their diagnoses were assessed against the confirmed diagnoses of those patients. A significant difference in favor of a UTE was found in some conditions, but not in those where the effect was expected to be larger (i.e., in complex problems). In other conditions, the difference was non-significant or even reversed. Overall, the results of the experiment were largely inconsistent with the predictions of the UTE theory.

Bonke et al. (2014) explored the UTE in a medical prognosis task. Both physicians and medical students were asked to estimate the life expectancy of four hypothetical patients. Accuracy was assessed by measuring the rank correlation between participants responses and the estimated life expectancy based on the number of favorable and unfavorable symptoms of each case. Case difficulty was manipulated experimentally. Half the participants were asked to spend some minutes thinking about the cases before making their diagnoses, while the remaining participants were asked to solve anagrams during that time. Although the descriptive statistics suggest that a UTE may have been present in some conditions, the main effect of thinking mode (UT vs. CT) was not statistically significant.

Finally, in a recent study by Woolley et al. (2015), family physicians were asked to diagnose three difficult cases based on real patients. Accuracy was measured against the patient’s known diagnosis (strict measure), and also against each case’s plausible diagnoses (lenient measure). Some physicians diagnosed immediately after reading each case, others after spending a few minutes performing a distracting *n*-back task, while others were asked to deliberate for as long as they needed. The study found no significant differences in diagnostic accuracy between the participants who were distracted before diagnosing and those in the two control groups, thus failing to replicate the UTE. To the best of our knowledge, these are the only studies that have explored the UTE in clinicians using a medical task that required domain knowledge.

In summary, the experiments conducted so far to explore the UTE in clinicians’ decision making have yielded contradictory results, with the effect being observed in the original report by de Vries et al. (2010) and in some (but not all) conditions of the Mamede et al. (2010) and Bonke et al. (2014) studies, but not in the Woolley et al. (2015) study. Although this suggests that the effect might not be reliable, alternative explanations are possible. First, the statistical power of these experiments might not be large enough to find a significant effect in all cases. Second, previous failures to replicate the UTE in other domains have been attributed to the potential contribution of a series of moderators (Strick et al., 2011). Perhaps the same moderators have prevented the effect from manifesting in these medical decision making studies. A simple approach to decide between these alternative interpretations is to conduct a meta-analysis on all the evidence available so far. If failures to replicate were simply due to a lack of statistical power, then a highly powered meta-analysis conducted on the results of all the experiments should yield a clear UT advantage. The potential role of moderators can also be assessed by means of meta-regressions.

Ideally, the effect sizes included in a meta-analysis should be statistically independent from each other. This assumption is violated when the original studies include several manipulations within one experiment, when researchers report the impact of a manipulation on several dependent measures, or when a specific group of participants (e.g., a UT condition) is compared with two different control groups (e.g., a CT and an immediate condition). A typical solution is to combine all the effect sizes from a particular study or condition into a single composite effect size (Nieuwenstein et al., 2015). However, this correction comes at a cost, because it involves losing any information about potential moderators that were manipulated within those conditions or experiments. Previous meta-analyses of the UTE have sometimes retained non-independent effect sizes when they conveyed valuable information about potential moderators (Strick et al., 2011). In the present review, we adopted both approaches. First, we conducted a meta-analysis including all data points. This allowed us to explore the role of a series of potential moderators. To make sure that the conclusions of the first meta-analysis were not biased by the non-independence of effect sizes, we conducted a second meta-analysis, where composite effect sizes were computed for non-independent conditions.

A key assumption of the UTE theory is that UT yields better decisions than CT only for complex decisions. Consistent with this assumption, a previous meta-analysis of the UTE found that the complexity of the task was a significant moderator of the effect (Strick et al., 2011). Dijksterhuis et al. (2009) suggested that the UTE is stronger with expert participants (but see González-Vallejo and Phillips, 2010). On the basis of this, it can be predicted that the UTE should be stronger in physicians than in medical students. These two factors (task difficulty and participants’ experience) were included as moderators in the meta-analysis. Similarly, Strick et al. (2011) found that the size of the UTE varies depending on the type of distracting task that participants in the UT condition are asked to perform. Specifically, the UTE was larger when participants were asked to conduct a word-search task, slightly smaller when they were asked to conduct an *n*-back task and smallest when they had to complete anagrams. In our first meta-analysis, this potential moderator was coded as a three-level variable.

We also included as potential moderators two features that have been manipulated in UTE experiments with medical decision tasks or that have varied substantially from one experiment to another. First, Mamede et al. (2010) and Woolley et al. (2015) used two different controls to assess the UTE: a CT condition, where participants responded after conscious deliberation, and an immediate condition, where participants responded immediately after the materials were presented. Some authors have argued that the UTE might actually be the product of poor performance in the CT conditions and that an immediate condition is a better control for UTE experiments (Shanks, 2006; Waroquier et al., 2010). Secondly, although all other experiments used a between-participants design, Mamede et al. (2010) manipulated thinking modality within participants. As these study features (type of control condition and study design) might play a role in the final effect size, we included them as moderators in the first meta-analysis.

The effect size of de Vries et al. (2010) was computed from the *F*-value reported in the main text. In the case of Mamede et al. (2010), the first author provided us with the descriptive statistics necessary to compute *d_av_* scores^1^. Effect sizes for Bonke et al. (2014) were computed from the descriptive statistics provided in their Table 4. In the case of Woolley et al. (2015), we had access to the raw data, which allowed us to compute different effect sizes for each control condition (CT and immediate) and for two levels of difficulty (Cases 1 and 2 were coded as complex and Case 3 as simple, based on participants’ mean accuracy rates of 18, 21, and 42%, respectively). These effect sizes were submitted to random-effects and mixed-effects meta-analyses using the metafor R package (Viechtbauer, 2010).

The results of the first meta-analysis are shown in **Figure 1**. As can be seen in the bottom row, the confidence interval of the random-effects model includes zero. Therefore the average effect size of these studies cannot be considered statistically significant, *z* = 0.35, *p* = 0.73. Analysis of heterogeneity showed that there were systematic differences across studies, *I^2^* = 69.90%, *Q*(16) = 51.83, *p* < 0.0001, possibly arising from the diversity of procedures, samples, and analysis strategies. The problem of heterogeneity is somewhat ameliorated by the use of a random-effects meta-analysis, which does not assume that all the studies are exploring exactly the same effect and which has a different interpretation from a fixed-effects meta-analysis (Riley et al., 2011).

**FIGURE 1:**
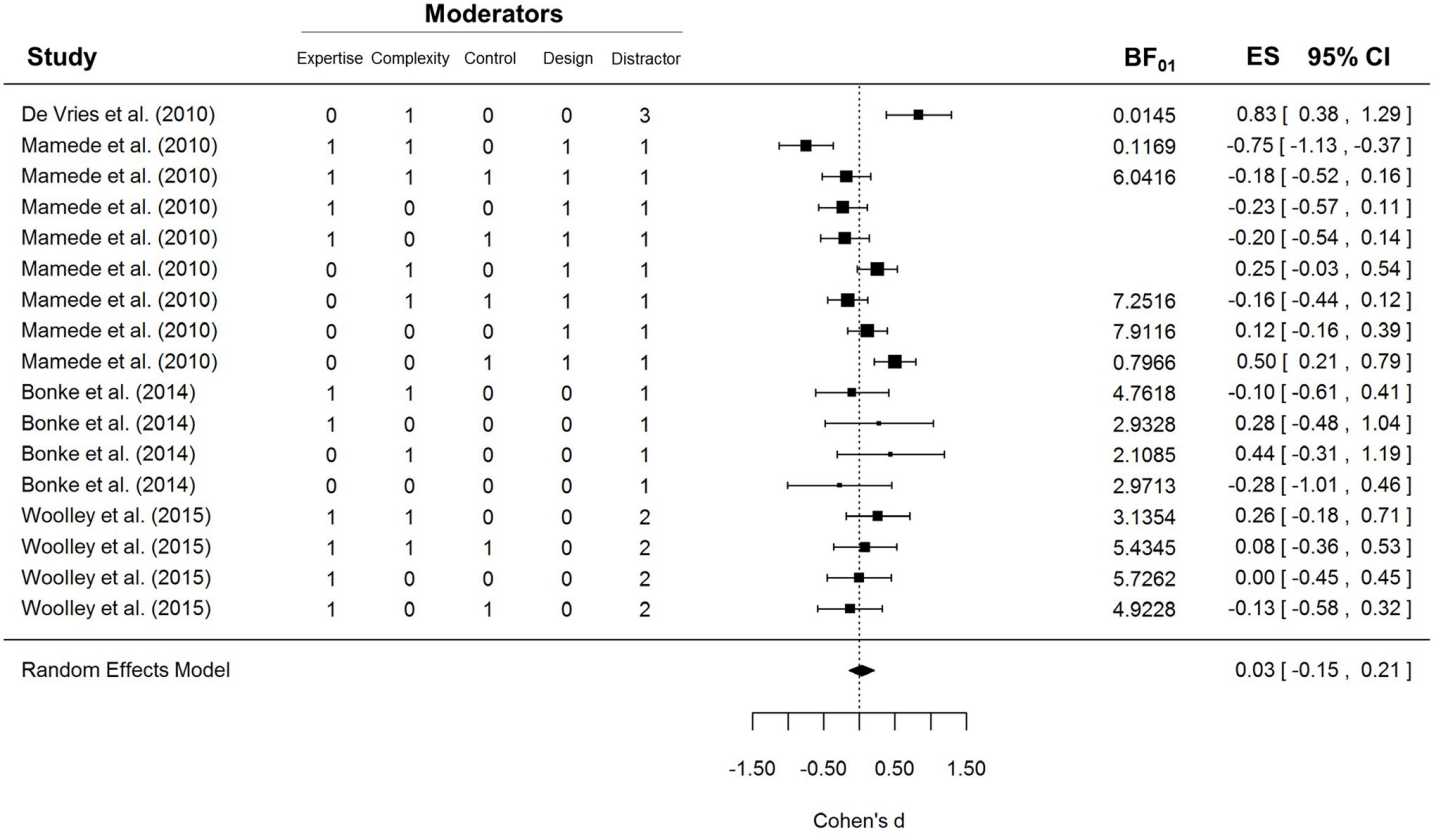
**Forest plot of the first meta-analysis**. Five moderators were included: Expertise, i.e., whether participants were novices (0) or experts (1); Complexity, i.e., whether the case materials were simple (0) or complex (1); Control, i.e., whether the UT condition was compared to a conscious-thought (0) or an immediate condition (1); Design, i.e., whether the study design was between- (0) or within-participants (1); and Distractor, i.e., whether the distraction task in the UT condition was anagrams (1), an *n*-back task (2), or a word-search puzzle (3).

To explore whether any of the five moderators could explain a proportion of the heterogeneity, we analyzed their impact by fitting an independent mixed-effects model for each one. Only expertise was a significant moderator, *Q*(1) = 5.71, *p* = 0.0169, but in the opposite direction to that suggested by Dijksterhuis et al. (2009): UT seemed to impair experts’ performance, *d* = -0.14, 95% CI [-0.32, 0.04], *Q*(9) = 15.77, *p* = 0.0718, and improve novices’ performance, *d* = 0.24, 95% CI [-0.02, 0.51], *Q*(6) = 20.23, *p* = 0.0025. The type of distractor task also approached significance, *Q*(1) = 3.83, *p* = 0.0503, but further inspection of the data shows that this effect is entirely driven by a single study that used a word-search puzzle. If the de Vries et al. (2010) study is removed from the sample, the moderating effect of the distractor task drops to negligible levels, *Q*(1) = 0.2278, *p* = 0.63. None of the other potential moderators reached statistical significance.

A critical advocate of the UT theory might suggest that the null result of the meta-analysis is due to publication bias: skeptical researchers might be more willing to publish the results of studies showing that UT does not improve medical decision making, and less willing to publish successful ones. If that were the case, then the results of the meta-analysis would be biased in favor of the null hypothesis and would not provide an accurate estimate of the true average effect size. Funnel plots provide a simple means to explore potential publication biases. If the effect sizes of the studies included in the meta-analysis are plotted against their standard errors, then the data points should appear scattered in a triangle-shaped distribution, where the most precise experiments would yield very similar estimations, while more variability would be observed in the less precise studies. If there is a publication bias, the regions of the funnel plot that correspond to non-published studies will be empty and, consequently, the funnel plot will not be symmetric.

**Figure 2** depicts the funnel plot of the effect sizes included in our first meta-analysis. As can be seen, all the data points seem to be distributed symmetrically around the vertical axis. Not surprisingly, a regression test for funnel plot asymmetry failed to find a significant effect, *t*(15) = 0.10, *p* = 0.92. Similarly, a trim-and-fill analysis (Duval and Tweedie, 2000) did not find studies missing on either side of the funnel plot. There is therefore no evidence of either positive or negative publication bias in the meta-analysis.

**FIGURE 2:**
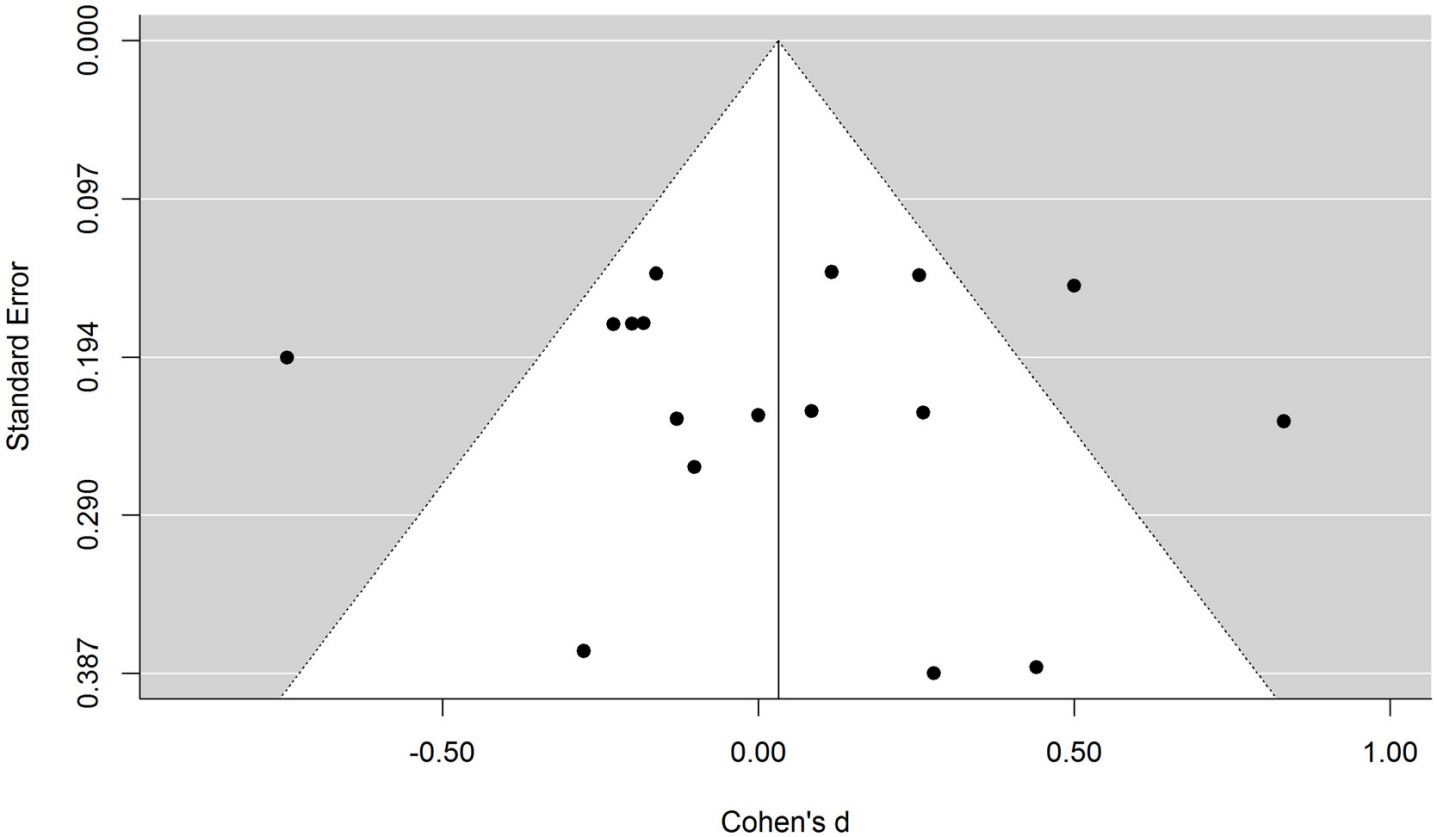
**Funnel plot of the first-meta-analysis**. Each data point represents the effect size and the standard error of one experimental condition. The white area represents a pseudo confidence-interval region around the effect-size estimate with bounds equal to ±1.96 standard error.

The forest plot in **Figure 1** shows that the UT effect was not significant in most conditions included in the meta-analysis. However, an important limitation of null-hypothesis significance testing (NHST) is that non-significant results are ambiguous: they can indicate either that the null hypothesis is true or that the data are not sensitive enough to provide clear support for the alternative hypothesis. Therefore, the non-significant results depicted in **Figure 1** cannot be taken as strong evidence that the UTE was absent in those experiments. Unlike NHST, Bayes factors (*BF_01_*) allow researchers to draw conclusions about null results (Rouder et al., 2009). To obtain a clearer insight into the null results of the studies included in the meta-analysis, we computed the *BF_01_*’s for each contrast using the BayesFactor R package. The *BF_01_* quantifies the extent to which each result is more consistent with the null hypothesis than with a generic alternative hypothesis; in this case, a Cauchy distribution with the scaling factor set to 1. This is a rather conservative prior that allows the alternative hypothesis to gather support from very small and even negative effect sizes. A *BF_01_* of 3 indicates that the data are three times more likely given the null hypothesis than the alternative hypothesis. Similarly, values below 1 indicate that the results are more likely given the alternative than the null hypothesis. Typically, values above 3 are considered substantial evidence for the null hypothesis and values below 0.33 are considered substantial evidence for the alternative hypothesis. *BF_01_*’s could not be computed for three conditions in Mamede et al. (2010) because exact *t*-values were not reported. As can be seen in **Figure 1**, most of the conditions included in the meta-analyses yielded *BF_01_*’s well above 1. Only three conditions yielded substantial evidence for the UTE, while one condition from Mamede et al. (2010) yielded substantial support for a CT advantage.

As mentioned earlier, an important shortcoming of this meta-analysis is that many of the conditions included in **Figure 1** are not independent of each other. To check that the overall results are not biased by the non-independence of data, we conducted a second meta-analysis including only independent effect sizes. A composite effect size was computed for all the related conditions in the Mamede et al. (2010) and Bonke et al. (2014) studies. Similarly, we computed a new effect size for Woolley et al. (2015) collapsing data across the three cases (i.e., the difficulty factor) and the two control conditions (CT and immediate). The results of the second meta-analysis are depicted in Supplementary Figure S1. Although the effect size estimate is somewhat less precise, *d* = 0.14, 95% CI [-0.16, 0.43], the main conclusions remain the same as in the first meta-analysis: the average effect of the random-effects model was not significantly different from zero, *z* = 0.92, *p* = 0.36, and the heterogeneity of results remained large and significant, *I^2^* = 71.16%, *Q*(5) = 16.36, *p* = 0.0059.

Overall, the results of our analyses suggest that the advantage of UT over conscious deliberation in medical decision making is an unreliable effect and that the failure of many experiments to replicate it reflects a genuine null result. It is unlikely that these negative results are due to insufficient power or to the presence of potential moderators. The only moderator that explained a proportion of the variance was expertise but in a direction opposite to that found by Dijksterhuis et al. (2009). Although a former meta-analysis of the wider literature found evidence in favor of the UTE (Strick et al., 2011), it is interesting to note that two other systematic reviews addressing this effect have yielded negative results (Acker, 2008; Nieuwenstein et al., 2015). The comprehensive meta-analysis recently conducted by Nieuwenstein et al. (2015) concentrated on multi-attribute choice and found no evidence for a UTE. Our results confirm that this also holds for the domain of professional medical decisions.

We should also note the failure of deliberation, as operationalized in the included studies, to produce consistently better medical decisions than the other two thinking modes (Bargh, 2011). Woolley et al. (2015) hypothesized that physicians’ decisions are mostly based on fast and efficient processes and online inferences that take place while information is being encoded (see also Flores et al., 2014). In fact, the median thinking time in their CT condition was only 7 s, suggesting that participants felt little need for further deliberation after reading the materials. If most of the decision making process takes place online, it is hardly surprising that neither a period of distraction nor the availability of time for further deliberation have a noticeable effect on performance.

For the time being, nothing suggests that clinicians should rely on UT to improve their decisions. Nevertheless, given the relatively small number of available studies on this important issue, further research should be encouraged. One obvious need is for careful replications of the experimental conditions that reported reliable advantages of UT over CT. A further need is for high-powered studies that explore a wider range of materials in combination with different distractor tasks. Finally, different types of deliberation conditions could be pitted against UT, e.g., self-paced (as in Woolley et al., 2015), fixed (as in Bonke et al., 2014), and proceduralized (as in Mamede et al., 2010). Before clinicians are formally advised to engage in UT to improve their decisions, the UTE should be explored extensively in conditions more similar to real clinical practice (see Woolley et al., 2015). Based on the little evidence available, such a recommendation would be premature at best.

## Conflict of Interest Statement

The authors declare that the research was conducted in the absence of any commercial or financial relationships that could be construed as a potential conflict of interest.

## Footnotes

1 Instead of using the standard deviation of the differences between conditions, as in a normal *t-test* for paired measures and the *Cohen’s d_z_* computed from it, Cohen’s *d_av_* scores are based on the average mean standard deviation across conditions (see Lakens, 2013).
