# Supplementary material for: A critical review and meta-analysis of the unconscious thought effect in medical decision making
Source: Front Psychol. 2015 May 19;6:636. doi: 10.3389/fpsyg.2015.00636 (PMC4436803; doi:10.3389/fpsyg.2015.00636)
Supplement: Supplementary file 1 [file Image_1.PDF]

**SUPPLEMENTARY MATERIAL**

**A critical review and meta-analysis of the unconscious thought effect in medical decision making**

Miguel A. Vadillo, Olga Kostopoulou, & David R. Shanks

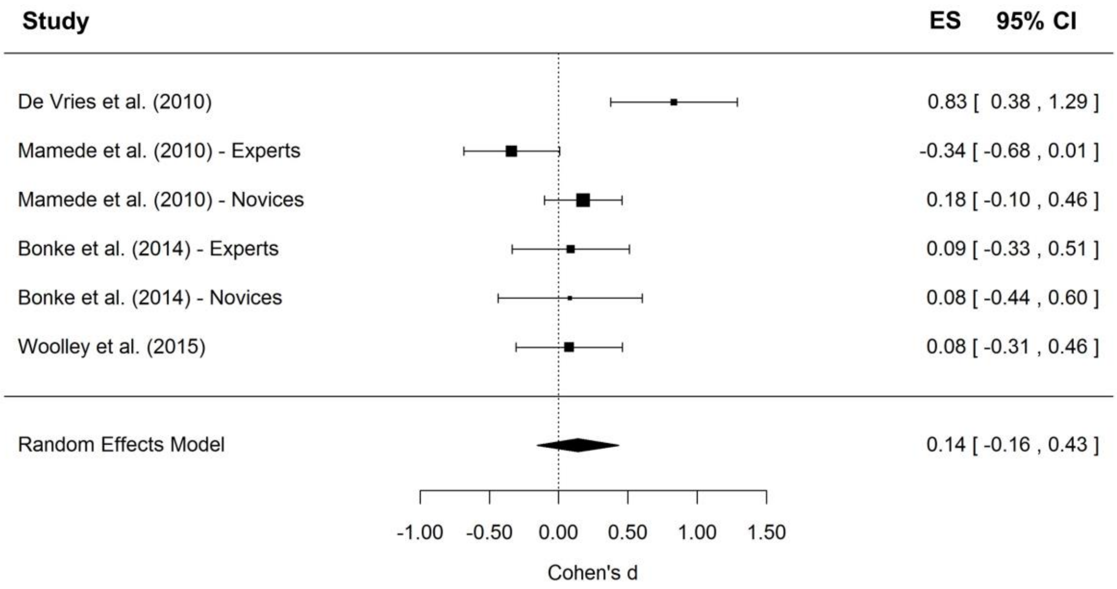


*Figure S1*. Forest plot of the second meta-analysis. Composite effect sizes were computed for Mamede et al. (2010) and Bonke et al. (2010). The effect size of Woolley et al. (2015) was computed from the raw data. The effect size of De Vries et al. (2010) is identical to the one included in the first meta-analysis. See the main text for more details.
